# Cost-effectiveness and cost-utility of COVID-19 vaccination in a South African privately insured population: a real-world claims-based analysis

**DOI:** 10.1186/s12913-026-15121-w

**Published:** 2026-07-20

**Authors:** Geetesh Solanki, Francesca Little, Susan Cleary

**Affiliations:** 1 Health Economics Unit, School of Public Health, University of Cape Town, Cape Town, South Africa; 2 NMG Consultants and Actuaries, Cape Town, South Africa; 3 Department of Statistical Sciences, University of Cape Town, Cape Town, South Africa

**Keywords:** COVID-19, Cost-effectiveness, Vaccination, South Africa, QALYs, Economic evaluation, Insurance claims, Markov model

## Abstract

**Background:**

COVID-19 placed strain on healthcare systems globally, including South Africa. While vaccine effectiveness is well established, context-specific evidence on the economic value of COVID-19 vaccination in African and real-world settings remains limited. This study evaluates the cost-effectiveness and cost-utility of COVID-19 vaccination within a South African population with private health insurance between 1 March 2020 and 30 December 2022 using claims data.

**Methods:**

A state-transition Markov model was developed in TreeAge Pro 2025 from a third-party payer perspective, comparing non-vaccination, partial vaccination, and full vaccination. Disease progression was simulated over a 60-year lifetime horizon using two-week cycles to capture acute and long-term health outcomes. Model inputs, including COVID-19-related hospitalisations, mortality, healthcare utilisation, and costs, were derived from insurance claims data for 550,332 individuals enrolled in two private health insurance funds during the study period. Outcomes included hospitalisations and deaths averted, life years (LYs) gained, and quality-adjusted life years (QALYs) gained. Costs and outcomes were discounted at 0% and 3% annually. Incremental cost-effectiveness and cost-utility ratios were calculated, and parameter uncertainty was assessed using one-way and probabilistic sensitivity analysis.

**Results:**

Both partial and full vaccination strategies were associated with fewer base case hospitalisations and deaths, minor gains in life years and QALYs, and lower lifetime discounted costs per individual at base, with $49.32 for non-vaccination, $23.32 for partial vaccination, and $49.08 for full vaccination. Partial vaccination was dominant compared to non-vaccination, while full vaccination was cost-saving at base case and in most simulations. Compared to partial vaccination, full vaccination provided little QALY gains at an incremental cost of $25.72 per individual, resulting in an ICUR of $64,424 per QALY gained.

**Conclusion:**

Using real-world insurance claims data, COVID-19 vaccination was associated with improved long-term health outcomes within a South African privately insured population, with partial vaccination being cost-saving. The second dose provided limited additional value in this lower-risk cohort. These findings indicate that funding vaccination during the pandemic represented an efficient allocation of healthcare resources within the insured sector. Caution is warranted in generalising these results beyond populations with different demographic and risk profiles.

## Introduction

### Background

COVID-19 was declared a pandemic by the World Health Organization (WHO) on 11 March 2020 [1, 2]. In South Africa, the first confirmed case was reported on 5 March 2020 [3], followed by a surge in infections that placed severe strain on the healthcare system, particularly in the public sector. The pandemic led to critical shortages in hospital beds, ICU capacity, medical supplies, and human resources [4], alongside significant increases in COVID-19-related hospital expenditures [5].

Globally, the economic costs of the pandemic have been estimated to range from US$77 billion to over US$2.7 trillion [6], with up to 4 million years of life lost (YLL) in high-incidence countries during the first year alone [7]. South Africa experienced similar economic disruption, with its economy contracting by at least 8% in 2020 [8].

In response to the global health crisis, vaccine development efforts accelerated rapidly, with several candidates entering clinical trials by mid-2020 [9]. South Africa launched its national COVID-19 vaccination programme in February 2021 [10]. According to the WHO COVID-19 dashboard, by 31 December 2023, South Africa had reported 4.1 million confirmed cases. Vaccination coverage stood at 70 doses administered per 100 people, with 41 people per 100 completing the primary vaccination series, and 7 per 100 receiving at least one booster dose [11].

International studies have shown that COVID-19 vaccination reduces hospitalisation [12–14], healthcare utilisation, and costs [15–17], with similar findings observed in South Africa [18–23]. However, while the clinical impact of vaccination is well-established, there is limited evidence on its cost-effectiveness using real-world data, particularly in African settings where access to healthcare services is limited, vaccine awareness varies, and suboptimal vaccine coverage, exacerbated by vaccine hesitancy, has posed challenges to achieving widespread protection [24]. In South Africa, this gap is especially important given the dual structure of the healthcare system and persistent resource constraints [25].

### Aim

This study evaluates the cost-effectiveness and cost-utility of COVID-19 vaccination within a South African population with private health insurance over the period 1 March 2020 to 30 December 2022, using real-world health insurance claims data. The objective is to inform health system financing and benefit design decisions by estimating the direct healthcare costs and long-term health outcomes associated with non-vaccination, partial and full vaccination strategies from a third-party payer perspective. Although the acute phase of the pandemic has ended, assessing the economic value of the strategies adopted during this period remains important for understanding the efficiency of health system responses in future public health emergencies.

## Methods

### Study design

This study follows the Consolidated Health Economic Evaluation Reporting Standards (CHEERS) 2022 guidelines for economic evaluations [26].

We conducted a modelled economic evaluation of COVID-19 vaccination based predominantly on data drawn from 550,332 individuals enrolled in two large South African private voluntary health insurance funds during the pandemic period [23]. The evaluation assesses direct healthcare costs, COVID-19 hospitalisations and deaths averted, Life Years (LYs) and Quality Adjusted Life Years (QALYs) gained, comparing three vaccination strategies: (1) Non-vaccination (baseline strategy), (2) Partial vaccination (one dose of a two-dose vaccine), and (3) Full vaccination (completion of a primary vaccination schedule). To reflect the time preference for costs and health outcomes, 0% and 3% annual discount rates were applied from the start of the simulation (cycle 0). The study was conducted from the insured payers perspective in which the cost to payers was based on the amounts paid by the health insurance funds to cover the claims submitted by health care providers for services rendered.

Incremental cost-effectiveness ratios (ICERs) were calculated per hospitalisation averted, per death averted and per LY gained. Incremental cost-utility ratios (ICURs) were calculated per QALY gained. The ratios were calculated as the difference in costs divided by the difference in outcomes of the different vaccination strategies. We assessed the strategies for dominance and for potential value for money by comparing the ICUR to the indicative cost effectiveness threshold (CET) of ZAR 38 500/ $2662.00 per DALY averted, representing the marginal productivity of the South African health system. Given the lack of alternative threshold, it is assumed that a DALY-based threshold can be used to understand the value for money of QALY-based ICURs [27]. All claims in South African Rands (ZAR) were converted to United States Dollar ($) values using the December $/ZAR exchange rate for the given year and then inflated/deflated to be expressed in 2022 values using the South African Consumer Price Index [28]. The model was solved using a probabilistic sensitivity analysis (PSA) with 10,000 iterations. This analysis enables an evaluation of uncertainty related to modelling parameters by randomly sampling values from each parameter distribution. This iteration count is consistent with standard practice for PSA in health economic evaluations and exceeds the number typically required for stable estimates.

For the PSA, triangular distributions were assigned to all parameters. For parameters derived from the claims data, 95% confidence intervals were first calculated using standard statistical methods. These interval bounds were then used as the minimum and maximum values of the triangular distribution, with the point estimate (mean) as the mode (see Table 1*).* The triangular distribution was selected for transparency and ease of implementation in TreeAge Pro. While alternative distributions (e.g., gamma for costs, beta for probabilities) are sometimes preferred, the triangular distribution is widely used in health economic evaluations and produces reliable mean estimates when parameters are well constrained by large sample sizes, as is the case in this study.

### Modelling approach

**Fig. 1:**
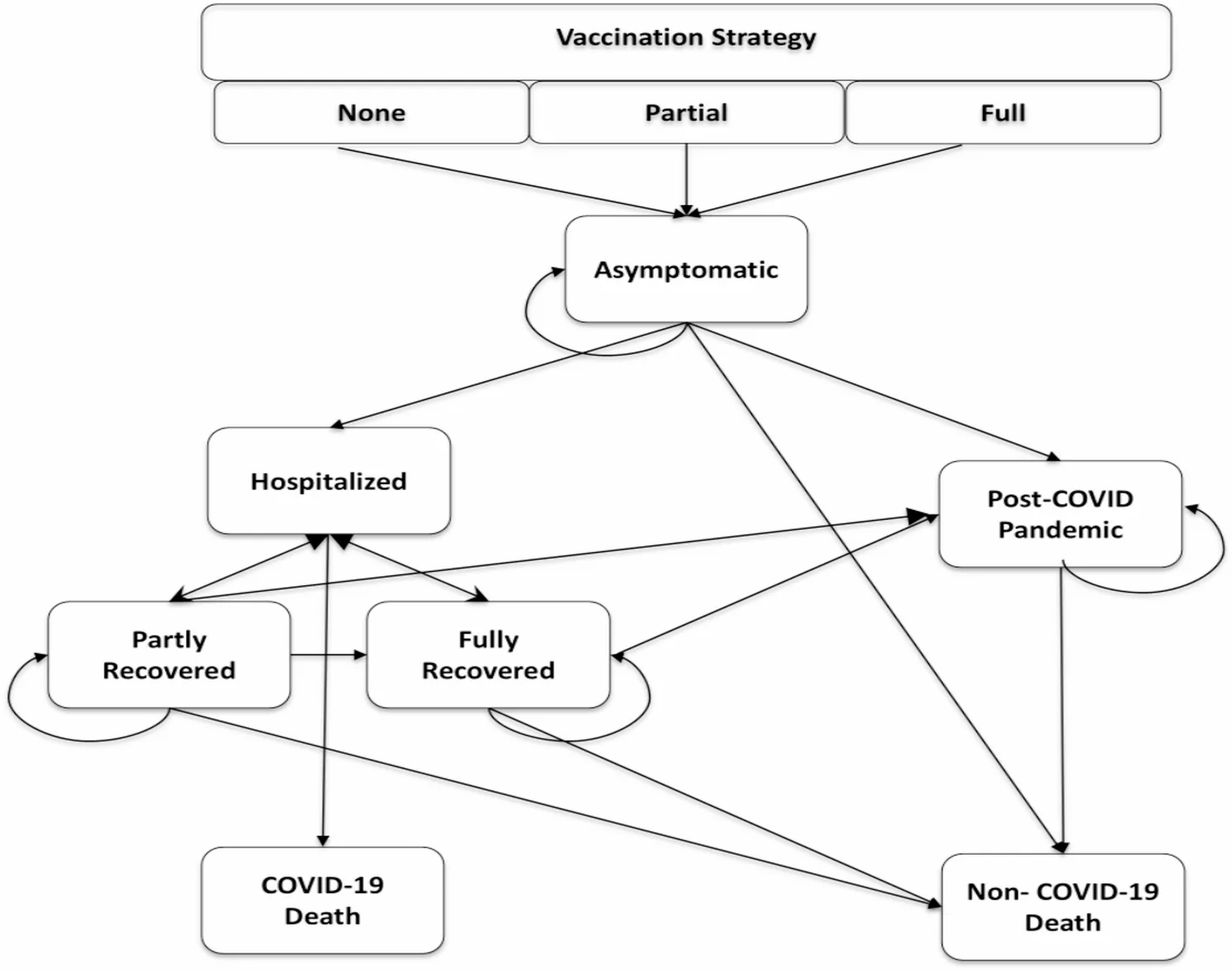
Decision analytic model structure

Three Markov models were developed using TreeAge Pro 2025 to compare alternative COVID-19 vaccination strategies: non-vaccination, partial vaccination, and full vaccination. The models have the same Markov state structure (Fig. 1), employ two-weekly Markov cycle lengths and run over a 60-year horizon - long enough to absorb all modelled individuals into the ‘Dead’ state and to capture the gains in life years or QALYs that may be associated with a vaccination strategy that averts COVID-19 related mortality. While the models have the same Markov state structure, they differ in terms of the costs and transition probabilities that are attached to the respective Markov states (as further described below and shown in Table 1). A full-cycle correction was applied to all cost and outcome reward sets.

At the start of the simulation, all individuals enter the model in the ‘Asymptomatic’ state. In the vaccinated groups, vaccine costs were applied as a once-off in this state. This approach ensures that no health events occur prior to vaccination, thereby avoiding immortal time bias in the comparison between vaccinated and non-vaccinated strategies. From there, individuals may remain asymptomatic, transition to the ‘Hospitalised’ state due to COVID-19 or die from other causes. We assume that those in ‘Asymptomatic’ do not die from COVID-19. Hospitalised individuals may transition to ‘Fully Recovered’, ‘Partly Recovered’ or ‘Dead’ (from COVID-19 or other causes). Those who are ‘Fully Recovered’ can remain in that state, be re-hospitalized, or die from other causes; they are assumed not to die from COVID-19. Individuals who are ‘Partly Recovered’ after ‘Hospitalized’ remain in this state for a limited number of cycles, ranging from 26 to 33, depending on vaccination status, before transitioning to ‘Fully Recovered’, returning to ‘Hospitalised’, or dying from COVID-19 or other causes. At the end of the pandemic period, all surviving individuals transition to the ‘Post Covid pandemic’ state where they remain until death from other causes. In all instances, death from other causes was applied in the model using age and sex specific per-cycle probabilities calculated from WHO life tables for South Africa [29], assuming an average age of 31.39 years at the start of the simulation, based on the average age in the study population at baseline.

While COVID-19 severity varies by age, the hospitalisation and mortality probabilities applied in the model were derived from real-world outcomes within the insured cohort and therefore reflect the overall risk profile of this population during the study period. Transition probabilities were not explicitly stratified by age group within the model structure, as such stratification was beyond the scope of this payer-focused evaluation, which aimed to estimate the average cost-effectiveness of vaccination in the insured population rather than age-specific effects. The model was verified by checking that extreme input values (e.g., zero vaccine efficacy, 100% hospitalisation probability) produced expected outputs, and by confirming that one-year model projections aligned with observed claims data.

A Markov modelling approach was selected for this study due to its transparency, suitability for modelling disease progression and long-term outcomes, and its widespread use in health economic evaluations of vaccination programmes [30]. The objective of this study was to evaluate the cost-effectiveness and cost-utility of COVID-19 vaccination within a defined insured population, rather than to model epidemic transmission dynamics at the population level. As such, indirect effects of vaccination through transmission reduction were not explicitly modelled. This approach is consistent with recent cost-effectiveness analyses that have adopted a payer perspective and utilized real-world claims data to estimate direct medical costs. While more complex microsimulation or dynamic transmission models may capture indirect epidemic effects, such approaches require assumptions regarding contact patterns and time-varying transmission parameters that were beyond the scope and objectives of this payer-focused evaluation [31, 32].

### Data sources

The core data for this modelling study were drawn from 550,332 individuals enrolled in two large South African private health insurance funds during the pandemic period - defined as 1 March 2020 to 30 December 2022. This period starts from the first reported COVID-19 case in South Africa to the end of the fourth wave and the removal of COVID-19 restrictions [33, 34]. The included population represents approximately 5% of the South African private health insurance sector and is comparable to the broader insured population in terms of family size, health insurance contributions, and healthcare expenditure [35]. Claims data for these individuals include inpatient and outpatient care, medication costs, and post-discharge follow-ups, enabling a comprehensive evaluation of direct medical costs. In addition, it is possible to estimate risk of hospitalisation, length of hospital stay, the probability of full recovery and the probability of death by COVID-19 vaccination status. In contrast, estimates relating to the risk of partial recovery following hospitalisation, duration in partly recovered states, and cost per day were sourced from peer-reviewed studies and applied to fit the study population [36–38]. Similarly, health related quality of life values to generate QALY estimates were also taken from the literature [39, 40].

### Data analysis

Detailed descriptions of the dataset and the methodology employed in extracting and analysing the claims data are provided in a prior publication [23]. Briefly, individuals in the claims dataset were allocated to one of the three vaccination strategies using an approach reported in the literature [41, 42]. The South African government authorized the Johnson and Johnson (J&J) and the Pfizer BioNTech vaccines. Individuals in the claims data who did not receive any vaccinations over the period of the pandemic were assigned to the non-vaccinated strategy. Individuals were allocated to the partly vaccinated strategy the day after their first vaccine dose (J&J or Pfizer BioNTech). Individuals were allocated to fully vaccinated 28 days after their first J&J vaccine or 14 days after their second dose of Pfizer BioNTech. Individuals receiving booster vaccinations remained fully vaccinated. COVID-19 vaccination costs included the claims from health care providers for services related to administering the vaccine and for the cost of the vaccine(s).

COVID-19 hospital admissions were identified using ICD-10 (10th revision of the International Statistical Classification of Diseases and Related Health Problems) code U07.1 or U07.2 [43]. Direct hospitalisation costs were then estimated by including all claims from health care providers for services rendered between the date of admission to the hospital to the date of discharge or death from the hospital. This included claims for accommodation, services, pharmaceuticals, medical and surgical consumables, and use of equipment from the hospital, specialists, auxiliaries, general practitioners and other primary care providers, and ambulance and advanced life support service providers. Treatment costs related to individuals with only partial recovery post discharge were derived using estimates from published literature and calibrated to the study population [36–38]. For the health-related quality of life estimates, values of 0.88 for the general population, 0.28 for those hospitalised with COVID-19 and 0.69 for those in a partly recovered state were used [39, 40]. The variables used in the model, together with the ranges on variables used for the probabilistic sensitivity analysis (PSA) are summarised in Table 1.

**Table 1:** Summary of model variables

	Non-vaccination	Partial vaccination	Full vaccination	Source
Cost: vaccination per person		$21.17 ($21.11,$21.22)	$47.64 ($47.55,$47.73)	[23]
Cost: per hospital day	$190.00 ($186.00, $195.00)	$155.00 ($149.00, $162.00)	$106.00 ($103.00, $110.00)	[23]
* Specialists*	*$90.05 ($87.90*,* $92.08)*	*$87.93 ($84.41*,* $91.60)*	*$69.19 ($67.04*,* $71.40)*	[23]
* Hospital*	*$77.07 ($75.22*,* $78.81)*	*$39.88 ($38.29*,* $41.55)*	*$18.92 ($18.33*,* $19.53)*	[23]
* Auxiliary*	*$15.70 ($15.32*,* $16.05)*	*$20.29 ($19.48*,* $21.14)*	*$14.04 ($13.60*,* $14.49)*	[23]
* GPs and related*	*$4.82 ($4.70*,* $4.93)*	*$4.86 ($4.66*,* $5.06)*	*$2.87 ($2.79*,* $2.97)*	[23]
* Ambulance/Advanced life support*	*$2.18 ($2.13*,* $2.23)*	*$2.03 ($1.94*,* $2.11)*	*$1.08 ($1.04*,* $1.11)*	[23]
* Support and other*	*$0.43 ($0.42*,* $0.44)*	*$0.27 ($0.26*,* $0.29)*	*$0.28 ($0.27*,* $0.29)*	[23]
Cost: per day part recovered	$0.45 ($0.44, $0.46)	$0.37 ($0.36, $0.39)	$0.25 ($0.25, $0.26)	[23, 37]
# Hospitalised/1,000/year	10.29 (9.73, 10.86)	0.67 (0.62, 0.72)	0.67 (0.62, 0.73)	[23]
Length of hospital stay (days)	9.19 (8.94, 9.45)	7.57 (6.92, 8.28)	7.35 (6.60, 8.19)	[23]
% hospitalised dying of COVID-19	19.25% (17.79%, 20.55%)	6.40% (5.96%, 6.87%)	5.43% (5.12%, 5.75%)	[23]
% hospitalised partly recovering	54.00% (44.00%, 63.00%)	34.00% (23.00%, 48.00%)	17.00% (12.00%, 22.00%)	[36, 44]
# of cycles partly recovered	32.60 (31.70, 33.50)	26.80 (24.50, 29.30)	26.10 (23.40, 29.00)	[23, 37]
RR* of dying: Partly recovered vs. gen. pop.	2.81 (1.48, 4.14)	1.87 (1.00, 2.75)	1.11 (1.00, 1.64)	[38]
All-cause mortality (all states)		Age- and sex-specific rates from WHO life tables applied in each cycle		[29, 45]
QALYs: General population		0.88(0.86,0.90)		[39]
QALYs: Hospitalised		0.28(0.26,0.30)		[39]
QALYs Partly recovered		0.69(0.67,0.71)		[39, 40]

Footnotes

• Vaccination costs are non-discounted per person; hospital and other costs are non-discounted per day

• RR: Relative risk

• All PSA distributions were triangular, defined using the reported mean as the mode and the confidence interval bounds as the minimum and maximum

• The ranges shown are the 95% confidence interval bounds, used as the minimum and maximum for the triangular distributions as a practical method of incorporating uncertainty into the PSA

As shown in Table 1, the cost of vaccination per individual was $21.17 for the partly vaccinated and $47.64 for the fully vaccinated. In terms of hospital costs, the cost per day, the length of stay, as well as the hospitalization events per 1000 individuals per year differ depending on vaccination status, with all estimates being highest in the non-vaccinated. There were also differences in the proportion dying in hospital, the proportion partly recovering post hospitalisation, the duration of time spent recovering, and the relative risk of dying during the recovery period, with worse outcomes in the non-vaccinated across all measures.

### Ethics

The data for the study on which a large part of this evaluation is based were accessed in terms of and under the conditions set out in the agreement between NMG and the client insurance funds and between NMG and the researcher. The terms set out in these agreements ensured that the access and use of the data is in full compliance with the requirements of the Protection of Personal Information (PoPI) Act in South Africa. Ethics approval for the use of the database to conduct this study was granted by the Human Research Ethics Committee of the Faculty of Health Sciences, University of Cape Town (reference number 606/2023).

### Results

Table 2 presents both 3.0% and 0.0% discounted base-case results, including costs (hospital, vaccination, and total) and outcomes (COVID-19 hospitalisations per 1,000 individuals, COVID-19 deaths, LYs and QALYs).

**Table 2:** Economic evaluation base case results for the vaccination strategies (discounted at 3% and 0%; in 2022 US$)

Strategy	Discount Rate: 3.0%		
	Non-vaccination (Baseline)	Partial vaccination	Full vaccination
**Dominance**	Abs. dominated	Undominated	Undominated
**Costs:**	$49.32 ($49.30, $49.35)	$23.32 ($23.32, $23.33)	$49.08 ($49.08, $49.08)
Cost: Hospitalisation	$49.30 ($49.27, $49.32)	$2.15 ($2.15, $2.15)	$1.44 ($1.44, $1.44)
Cost: Vaccination	$0.00 ($0.00, $0.00)	$21.17 ($21.17, $21.17)	$47.64 ($47.64, $47.64)
**Outcomes:**			
COVID Hospitalisation/1000	28.204 (28.191, 28.216)	1.827 (1.826, 1.829)	1.837 (1.836, 1.838)
COVID Deaths/1000	5.410 (5.406, 5.414)	0.117 (0.117, 0.118)	0.100 (0.100, 0.100)
LYs	21.640 (21.640, 21.640)	21.753 (21.753, 21.753)	21.754 (21.754, 21.754)
QALYs	19.039 (19.035, 19.042)	19.143 (19.139, 19.146)	19.143 (19.139, 19.146)
	Discount Rate: 0.0%		
**Strategy**	Non-vaccination (Baseline)	Partial vaccination	Full vaccination
**Dominance**	Abs. dominated	Undominated	Undominated
**Costs:**	$51.49 ($51.47, $51.52)	$23.42 ($23.41, $23.42)	$49.14 ($49.14, $49.15)
Cost: Hospitalisation	$51.45 ($51.43, $51.48)	$2.24 ($2.24, $2.25)	$1.50 ($1.50, $1.51)
Cost: Vaccination	$0.00 ($0.00, $0.00)	$21.17 ($21.17, $21.17)	$47.64 ($47.64, $47.64)
**Outcomes:**			
COVID Hospitalisation/1000	29.413 (29.400, 29.426)	1.905 (1.904, 1.907)	1.916 (1.915, 1.917)
COVID Deaths/1000	5.647 (5.643, 5.652)	0.123 (0.123, 0.123)	0.104 (0.104, 0.104)
LYs	39.263 (39.263, 39.263)	39.475 (39.475, 39.475)	39.476 (39.476, 39.476)
QALYs	34.543 (34.537, 34.549)	34.734 (34.727, 34.740)	34.734 (34.728, 34.741)

Footnotes

• All costs in this table refer to discounted lifetime present values (0% and 3% annually)

•  Values in parentheses are 95% uncertainty intervals from the probabilistic sensitivity analysis (percentile method)

•  QALY gains for partial and full vaccination are identical when shown to 3 decimal places. At full precision, full vaccination provides a small additional QALY gain of 0.0004 (3% discount) compared to partial vaccination

As the 3.0% and 0.0% discounted results followed similar patterns, only the 3.0% discounted results are discussed. The 3.0% discounted lifetime hospitalisation cost per individual was estimated at $49.30 for the non-vaccinated group, compared to $2.15 for the partly vaccinated and $1.44 for the fully vaccinated. Vaccination costs were $23.32 for the partly vaccinated and $49.08 for the fully vaccinated. Accordingly, total 3.0% discounted lifetime costs were $49.32 for the non-vaccinated, $23.32 for the partly vaccinated, and $49.08 for the fully vaccinated.

Hospitalisation rates were 28.204 per 1,000 individuals for the non-vaccinated, compared to 1.827 and 1.837 per 1,000 for the partly and fully vaccinated, respectively. COVID-19 mortality rates were 5.410 per 1,000 for the non-vaccinated, 0.117 for the partly vaccinated, and 0.100 for the fully vaccinated. The 3.0% discounted LYs were 21.640 for non-vaccinated individuals, 21.753 for the partly vaccinated and 21.754 for the fully vaccinated. The 3.0% discounted QALYs were 19.039 for the non-vaccinated, 19.143 for the partly vaccinated and 19.143 for the fully vaccinated. Across all outcomes, including hospitalisations averted, COVID-19 deaths averted, LYs gained, and QALYs gained, both vaccination strategies were dominant compared to the non-vaccination baseline, yielding lower overall costs and improved health outcomes.

Table 3 presents both 3.0% and 0.0% discounted sequential comparison results, including incremental costs, incremental outcomes (COVID-19 hospitalisations averted per 1,000 individuals, COVID-19 deaths averted per thousand individuals, LYs gained, and QALYs gained), and the corresponding ICERs and ICURs for each comparison (Partial vs. Non-vaccination, Full vs. Non-vaccination, and Full vs. Partial vaccination).

**Table 3:** Incremental cost-effectiveness and cost-utility results for all pairwise comparisons (discounted at 3% and 0%; in 2022 US$)

Parameter	Discount Rate: 3.0%		
	Partial vaccination vs. Non-vaccination	Full vaccination vs. Non-vaccination	Full vaccination vs. Partial vaccination
Incremental Cost ($)	-26.00 (-26.03, -25.97)	-0.24 (-0.27, -0.21)	+ 25.76 (+ 25.76, + 25.76)
Δ Hospitalisations averted / 1,000	+ 26.377 (26.365, 26.388)	+ 26.367 (26.355, 26.378)	-0.010 (-0.012, -0.008)
ICER ($ per hospitalisation averted)	Dominant	Dominant	Dominated
Δ Deaths averted / 1,000	+ 5.293 (5.289, 5.297)	+ 5.310 (5.306, 5.314)	+ 0.017 (+ 0.015, + 0.019)
ICER ($ per death averted)	Dominant	Dominant	$1,515
Δ Life Years (LYs)	+ 0.113 (0.113, 0.113)	+ 0.114 (0.114, 0.114)	+ 0.001 (0.001, 0.001)
ICER ($ per LY gained)	Dominant	Dominant	$25,760
Δ QALYs	+ 0.104 (0.104, 0.104)	+ 0.104 (0.104, 0.104)	0.000 (0.000, 0.000)
ICUR ($ per QALY gained)	Dominant	Dominant	$64,424
	Discount Rate: 0.0%		
**Parameter**	Partial vaccination vs. Non-vaccination	Full vaccination vs. Non-vaccination	Full vaccination vs. Partial vaccination
Incremental Cost ($)	-28.07 (-28.06, -28.08)	-2.35 (-2.34, -2.36)	+ 25.72 (+ 25.72, + 25.72)
Δ Hospitalisations averted / 1,000	+ 27.508 (27.496, 27.520)	+ 27.497 (27.485, 27.509)	-0.011 (-0.013, -0.009)
ICER ($ per hospitalisation averted)	Dominant	Dominant	Dominated
Δ Deaths averted / 1,000	+ 5.524 (5.520, 5.528)	+ 5.543 (5.539, 5.547)	+ 0.019 (+ 0.017, + 0.021)
ICER ($ per death averted)	Dominant	Dominant	$1,354
Δ Life Years (LYs)	+ 0.212 (0.212, 0.212)	+ 0.213 (0.213, 0.213)	+ 0.001 (0.001, 0.001)
ICER ($ per LY gained)	Dominant	Dominant	$25,720
Δ QALYs	+ 0.191 (0.191, 0.192)	+ 0.191 (0.191, 0.192)	0.000 (0.000, 0.000)
ICUR ($ per QALY gained)	Dominant	Dominant	$64,300

Footnotes

• All costs in this table refer to discounted lifetime present values (0% and 3% annually)

• Values in parentheses are 95% uncertainty intervals from the probabilistic sensitivity analysis (percentile method)

• A strategy is considered dominant when it is both less costly and more effective than the comparator

• A strategy is considered dominated when it is more costly and less effective than the comparator

• Δ QALYs shown to 3 decimal places. At full precision, the incremental QALY gain is 0.0004 at both discount rates. Slight differences in the ICUR reflect rounding in discounted costs

Both partial and full vaccination strategies were dominant compared to the non-vaccination strategy (Table 3). As is shown, each vaccination strategy reduced costs and increased outcomes in comparison to non-vaccination. However, in comparison to partial vaccination, full vaccination increased costs with little benefit in terms of deaths averted, life years and QALYs gained, and a potential loss in terms of hospitalisations averted. The ICUR was $64,424 per QALY gained, reflecting the very small incremental QALY difference (0.0004 when calculated at full precision).

**Fig. 2:**
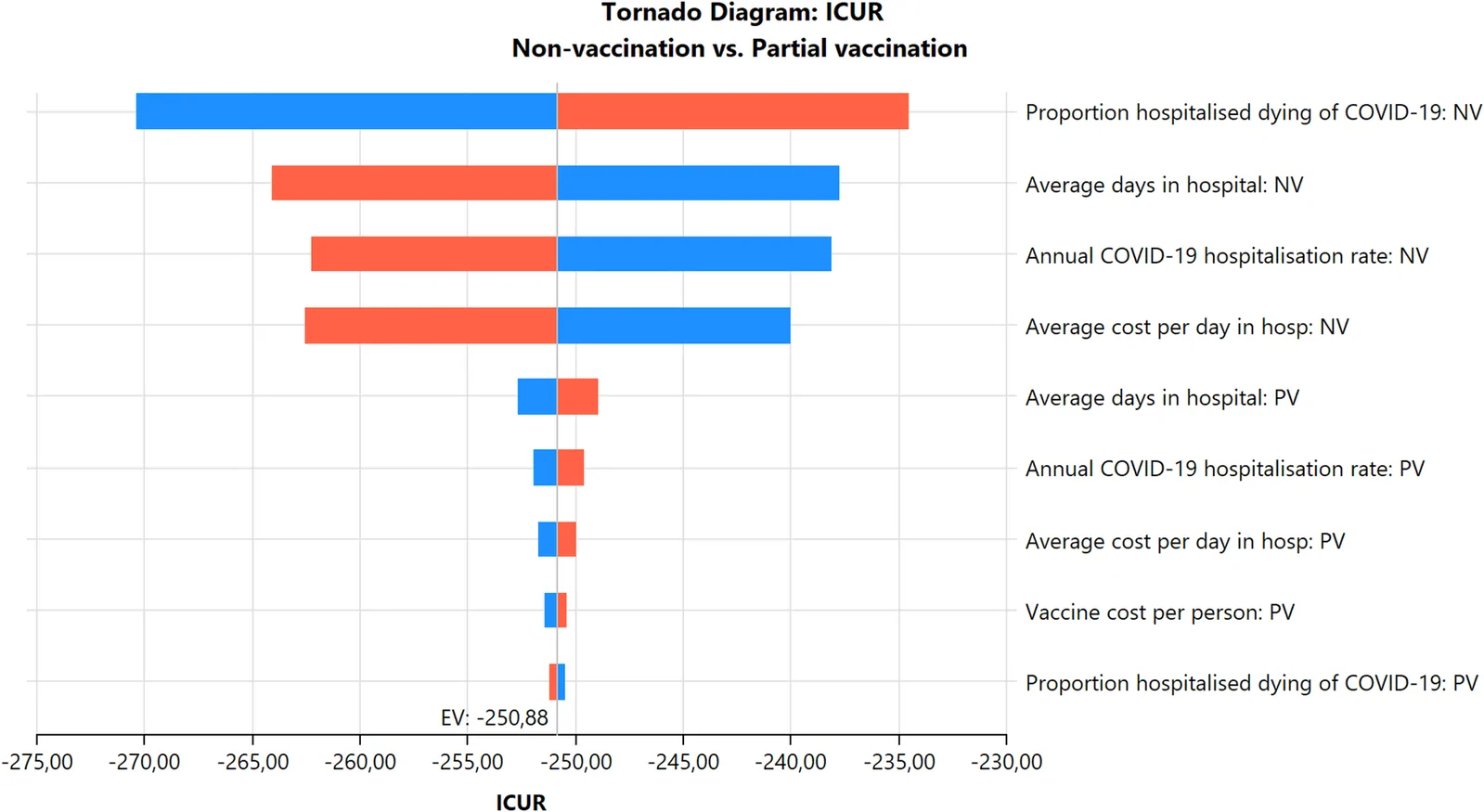
Tornado diagram: Non-vaccination vs. Partial vaccination (3% discount). Footnote: NV- Non-vaccinated, PV- Partly vaccinated, FV- fully vaccinated

**Fig. 3:**
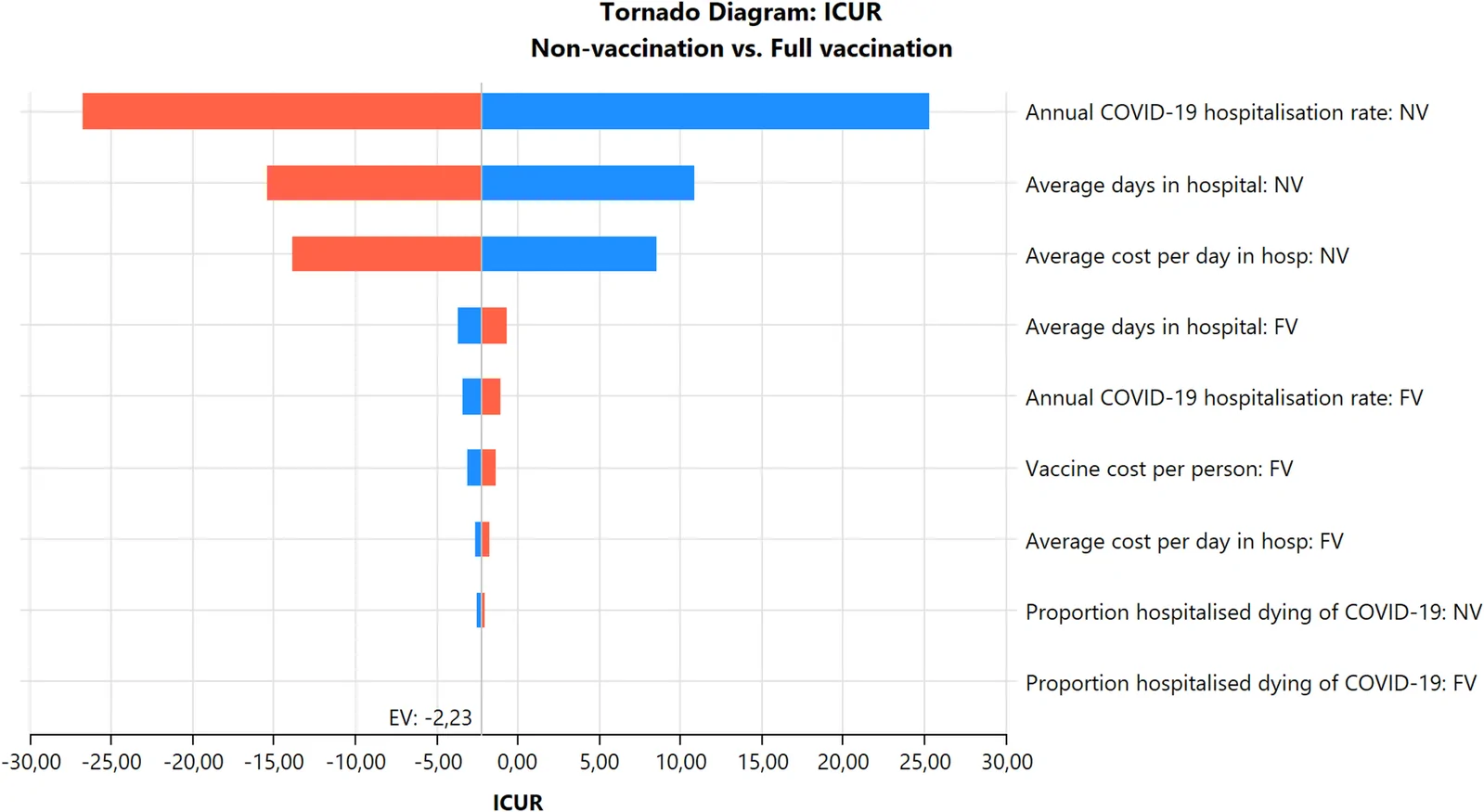
Tornado diagram: Non-vaccination vs. Full vaccination (3% discount). Footnote: NV- Non-vaccinated, PV- Partly vaccinated, FV- fully vaccinated

**Fig. 4:**
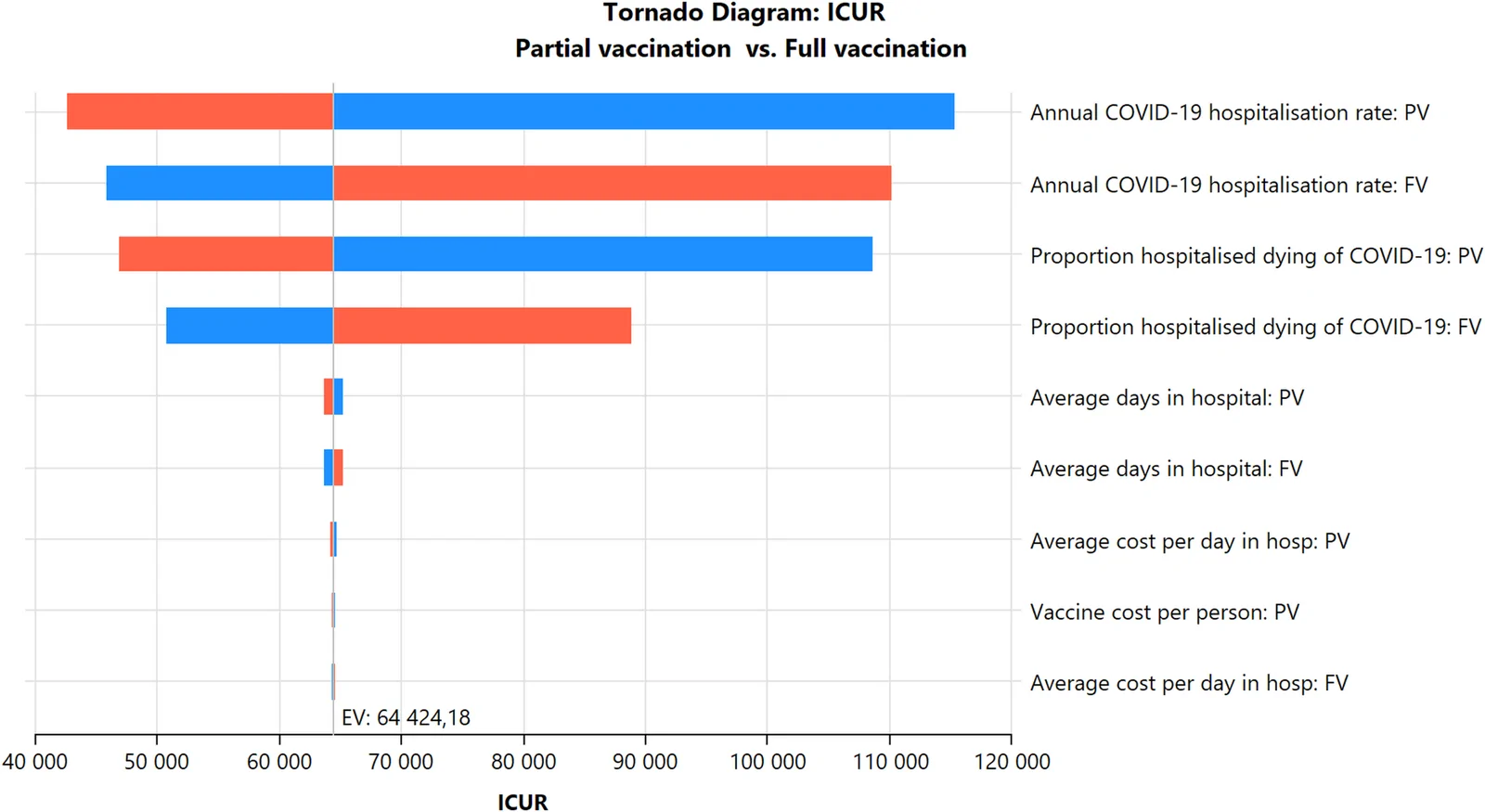
Tornado diagram: Partial vaccination vs. Full vaccination (3% discount)

One-way sensitivity analyses focused on the ICUR results (cost per QALY gained) (Figs. 2, 3 and 4). For partial vaccination vs. non‑vaccination (Fig. 2), the ICUR remained cost‑saving across all parameter variations. The most influential parameter was the proportion of non‑vaccinated hospitalised individuals dying of COVID‑19, followed by the average length of hospital stay and the hospitalisation rate among non‑vaccinated individuals.

For full vaccination vs. non‑vaccination (Fig. 3), the ICUR was cost‑saving at the base case, though some parameter variations yielded a positive ICUR. The most influential parameter was the hospitalisation rate for the non-vaccinated, followed by the average length of hospital stay and the cost per day in hospital among non-vaccinated individuals.

For full vaccination vs. partial vaccination (Fig. 4), the ICUR remained above the South African cost‑effectiveness threshold ($2,662 per DALY averted) across all parameter variations. The most influential parameters were the annual COVID‑19 hospitalisation rates among partly and fully vaccinated individuals and the proportion of partly vaccinated hospitalised individuals dying of COVID‑19.

**Fig. 5:**
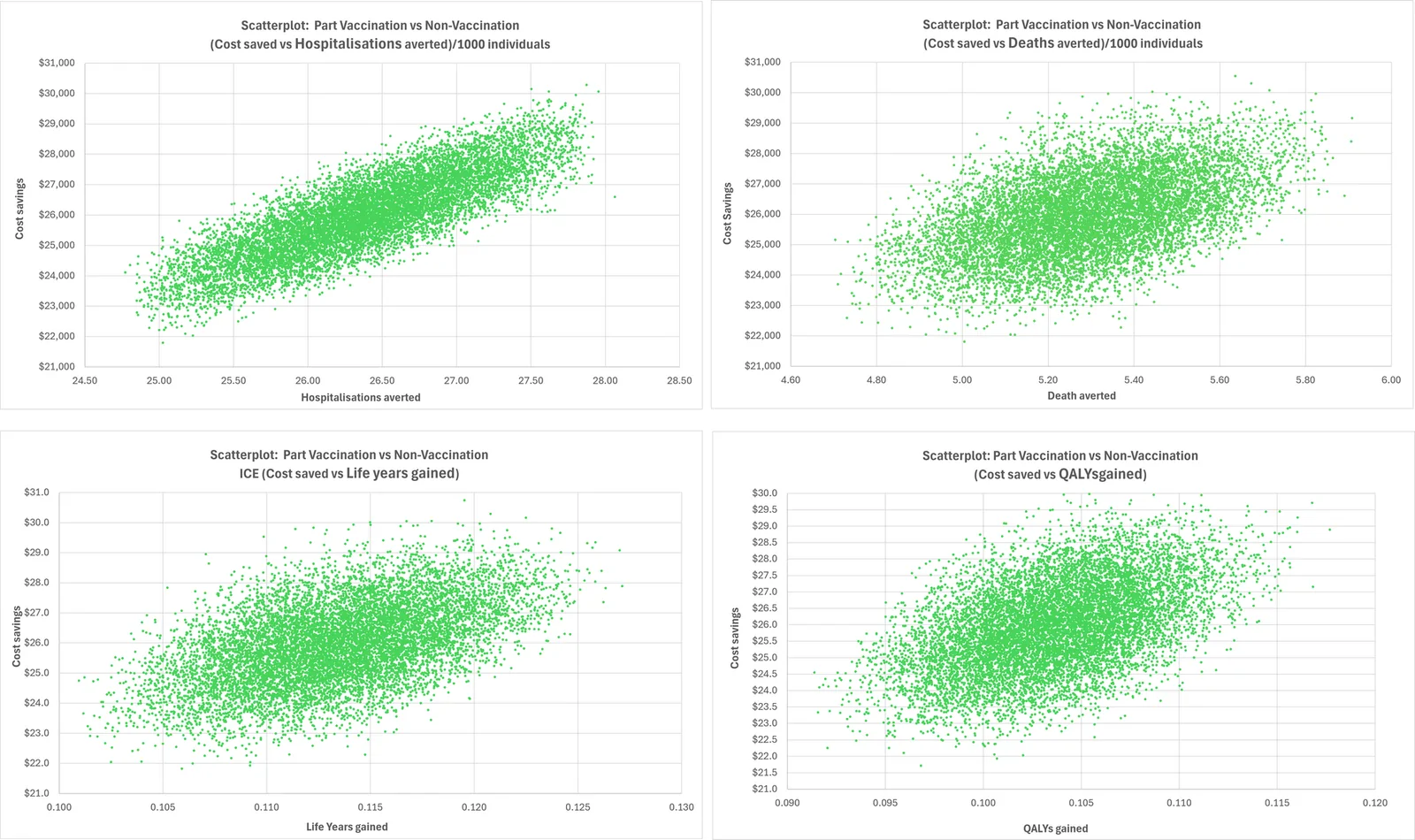
Sensitivity analysis Partial vaccination versus Non-vaccination: Scatterplots of incremental costs vs. incremental outcomes

**Fig. 6:**
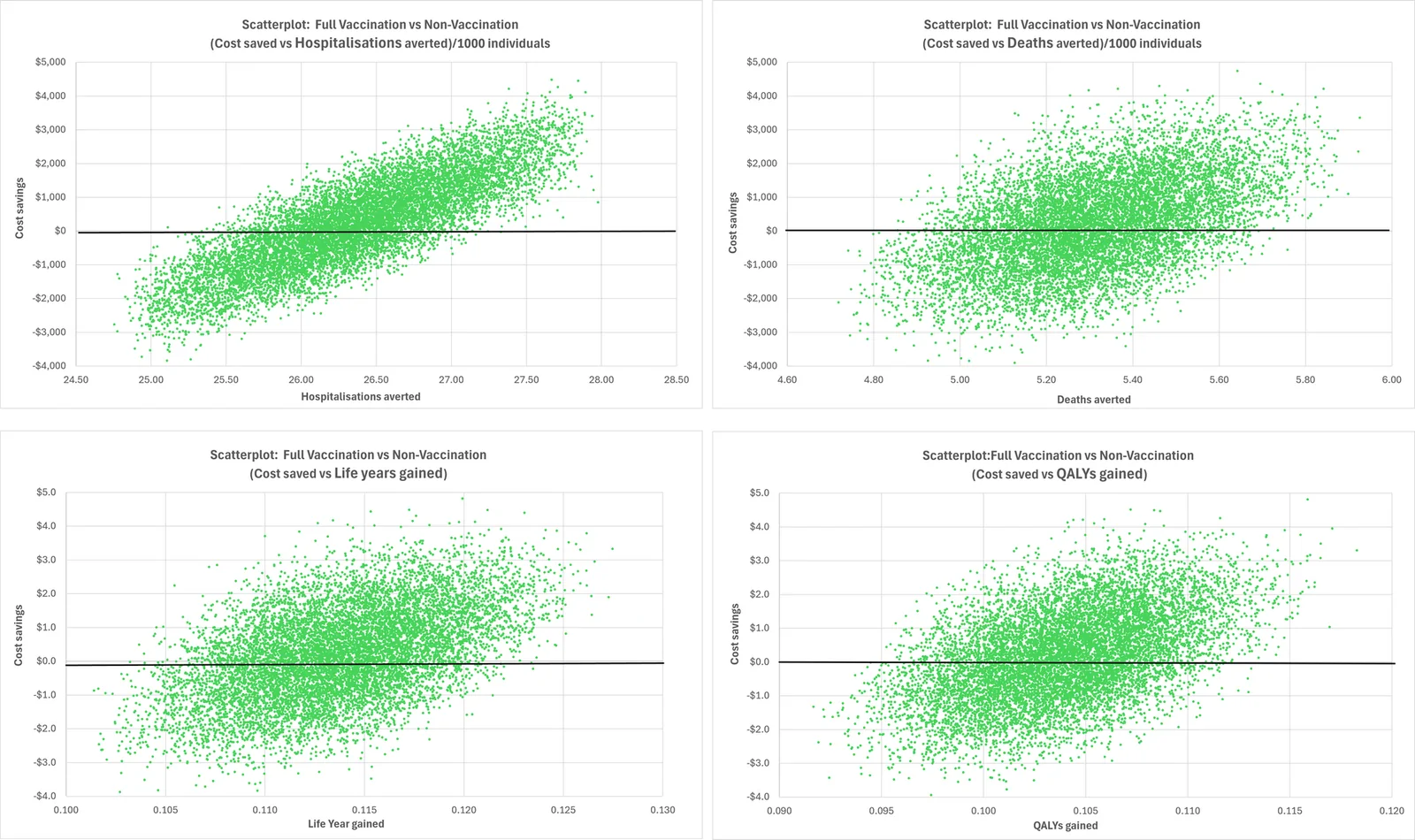
Sensitivity analysis Full vaccination vs. Non-vaccination: Scatterplots of incremental costs vs. incremental outcomes

**Fig. 7:**
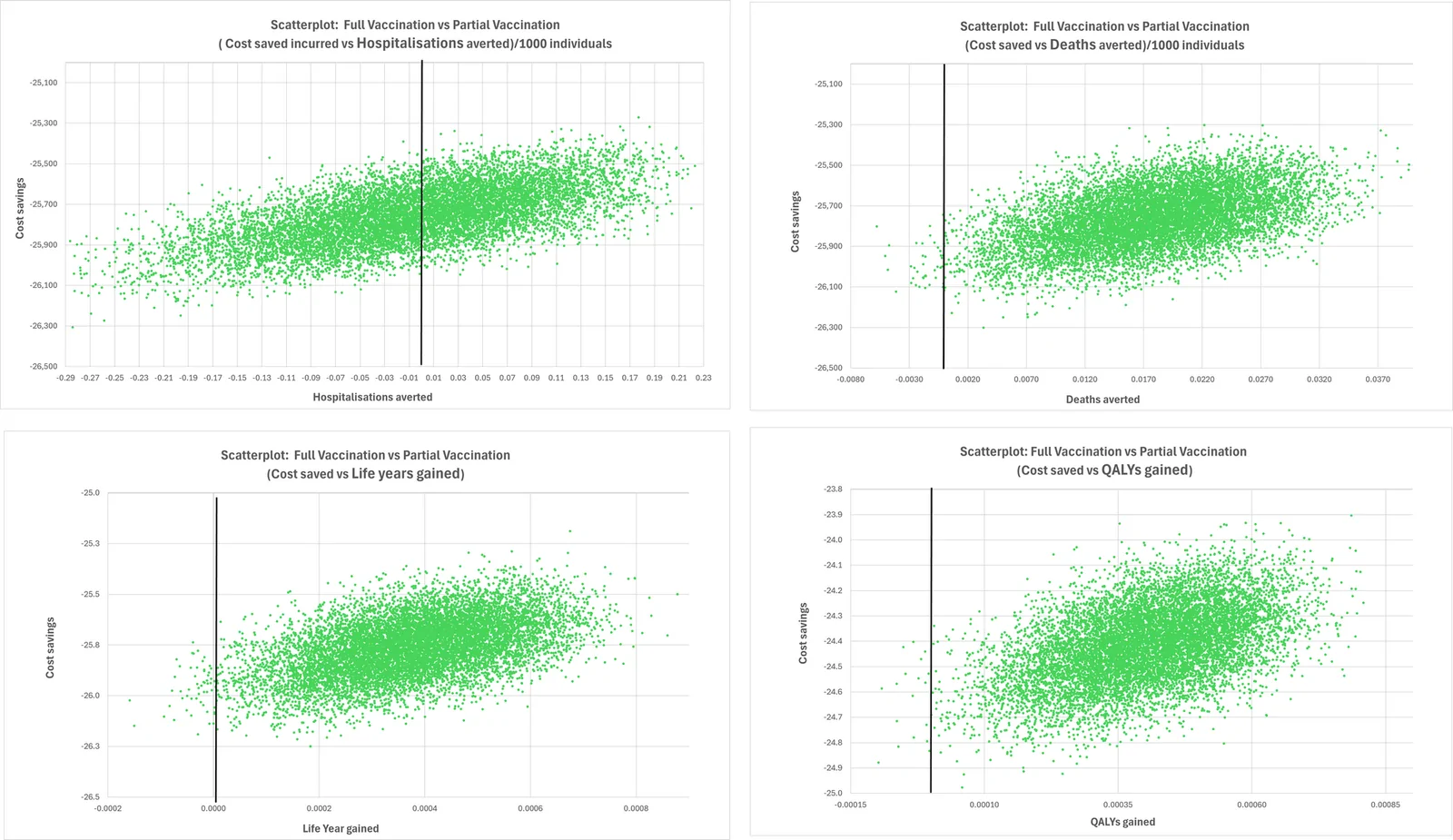
Sensitivity analysis Full vaccination vs. Partial vaccination: Scatterplots of incremental costs vs. incremental outcomes

The results of the probabilistic sensitivity analysis (PSA), showing scatterplots of incremental costs versus incremental effects for the four outcomes measured are presented in Fig. 5 for the partial vaccination versus non-vaccination strategies, Fig. 6 for the full vaccination versus non-vaccination strategies, and Fig. 7 for the full vaccination versus partial vaccination strategies.

To aid interpretation, the PSA scatterplots were plotted with the axes reversed with cost savings on the Y-axis; and adverse events averted (hospitalisations, deaths) together with health outcomes gained (LYs and QALYs) expressed on the X-axis. Under this orientation, points in the north-east quadrant indicate strategies that are both more effective and less costly than the comparator (i.e., absolutely dominant).

For the partial vaccination versus non-vaccination scatterplot (Fig. 5), all scatter points across all outcomes show cost savings and improved health outcomes, indicating that partial vaccination consistently outperformed non-vaccination.

For the fully vaccinated versus non-vaccinated scatterplot (Fig. 6), across all outcomes (hospitalisations averted, deaths averted, life-years gained, and QALYs gained), full vaccination was never associated with worse health outcomes compared to non-vaccination. Between 55.6% and 57.0% of simulations showed both cost savings and improved health outcomes, while the between 43.0% and 44.4% showed improved health outcomes at higher cost.

For the full vaccination versus partial vaccination scatterplot (Fig. 7), the pattern varied by outcome. For COVID-19 deaths averted, life years and QALYs gained, full vaccination was more effective but at higher cost in the vast majority of simulations (99.6%–99.8%), with a negligible fraction showing worse outcomes at higher cost. For hospitalisations averted, the points show consistently higher cost with no meaningful difference in hospitalisation rates, reflecting the similar hospitalisation rates between the two vaccinated groups.

## Discussion

The findings of this economic evaluation indicate that both partial and full COVID-19 vaccination strategies were more effective than non-vaccination within a large South African privately insured population during the pandemic period. Partial vaccination was cost-saving across all simulations, while full vaccination was cost-saving at the base case and in the majority of simulations (55.6%–57.0%), with the remainder showing improved outcomes at higher cost. Both strategies generated improved health outcomes, with vaccination costs offset by reductions in hospitalisation and severe disease, resulting in lower or comparable lifetime expenditure. Probabilistic sensitivity analysis confirmed the robustness of these findings, with all simulations for partial vaccination and the majority for full vaccination indicating improved outcomes.

The pattern emerging from a direct comparison of the two vaccination strategies was different. For the sequential comparison of full vaccination versus partial vaccination, the incremental cost-utility ratio (ICUR) was $64,424 per QALY gained. This exceeds the South African cost-effectiveness threshold of $2,662 per DALY averted. The very small incremental QALY gain (0.0004 at 3% discount) reflects the already low risk of severe COVID-19 among partially vaccinated individuals in this young, lower-risk insured population. The small negative value for hospitalisations averted in this comparison (-0.009 per 1,000 at 3% discount) reflects a marginal, not statistically significant increase in hospitalisations for fully vaccinated individuals compared to those partially vaccinated. This is more likely to be due to random variation rather than a true biological effect, given the extremely low hospitalisation rates in both vaccinated groups. As indicated by the PSA (Fig. 7), the hospitalisation scatter points show consistently higher cost with no meaningful difference in hospitalisation rates, confirming the near-identical hospitalisation rates between the two vaccinated groups. Taken together, these findings suggest that, from a payer perspective, the second dose provided little additional health benefit and did not represent good value for money in this specific demographic, although it remained highly cost-effective compared to no vaccination.

From a payer perspective, these findings provide empirical support for the funding of COVID-19 vaccination during the pandemic. The results indicate that vaccination not only reduced clinical burden but also alleviated pressure on hospital expenditure within the insured health system. The modest gains observed in LYs and QALYs reflect the relatively young age profile of this insured population, yet even within this lower-risk cohort, vaccination generated measurable long-term health benefits alongside immediate cost savings. However, the high ICUR for the second dose suggests that, under resource constraints, prioritising first doses may offer greater economic efficiency in young, low‑risk populations.

These findings are consistent with national and international evidence demonstrating the economic value of COVID-19 vaccination. Within South Africa, a prior evaluation by Edoka et al. of the national vaccination programme reported an incremental cost-effectiveness ratio of approximately US$1,300 per disability-adjusted life year averted, below the country’s indicative cost-effectiveness threshold of US$2,300 [46]. The apparent discrepancy between our finding of cost-saving (at base case) and Edoka et al.‘s positive ICER is likely to be due to key differences in study design. Edoka et al. evaluated the national public sector programme (older population, different vaccine mix, including rollout costs), while our study assesses a privately insured, younger population (mean age 31.4 years) from a payer perspective. The younger, lower-risk profile of our cohort results in smaller absolute cost savings, but dominance (negative incremental costs) remains, as vaccination reduces healthcare costs even in this lower-risk group. By contrast, a study based on a microsimulation model found that a vaccination strategy covering at least 40% of the population could prevent over 9 million infections and more than 73,000 deaths, while also reducing healthcare costs [32].

Internationally, at least three systematic reviews of economic evaluation of COVID-19 vaccination studies have reported comparable results. The first, a systematic review of 25 studies concluded “that COVID-19 vaccination was a cost-effective or cost-saving intervention for mitigating coronavirus transmission and its effect in many countries within certain conditions. Most studies reported vaccine efficacy values ranging from 65% to 75%”. The second, a review of 40 studies concluded that “COVID-19 vaccination is a cost-effective alternative in preventing and controlling COVID-19” [47]. The third, a review of economic evaluation of broader strategies against COVID-19 based on 36 studies, concluded that “all strategies are likely to be more cost-effective against COVID-19 than no intervention and vaccination was the most cost-effective strategy [48].”

This study contributes additional context-specific evidence derived from real-world claims data within a middle-income country health insurance setting. A key strength of this analysis is the use of detailed real-world claims data from over 550,000 individuals, allowing for empirically robust estimates of hospitalisation risk, length of stay, mortality, and direct medical costs by vaccination status. The lifetime horizon and inclusion of both cost-effectiveness and cost-utility outcomes provide an assessment of long-term value.

These findings have implications for pandemic preparedness financing in middle-income insurance systems. The observation that partial vaccination (one dose of a two-dose primary series) delivered most of the economic return in this relatively young, lower-risk population suggests that during future pandemics or vaccine supply constraints, prioritising first doses over second doses in younger, lower-risk populations may maximise healthcare cost savings from a payer perspective. However, full vaccination (completion of the primary series) remains important for older or medically vulnerable populations. The limited additional economic return from the second dose in this cohort also raises questions about booster strategies: in low-risk populations, the marginal economic benefit of additional doses beyond the primary series may be limited once baseline protection is established.

This study has several limitations. First, the analysis was based predominantly on data from a privately insured population and therefore does not fully reflect the demographic, socioeconomic, and risk profile of the broader South African population, particularly uninsured individuals who have different exposure risks and access to care [25]. The findings should therefore be interpreted primarily within the context of insured health system decision-making. Second, although hospitalisation and mortality probabilities were derived from real-world outcomes within the insured cohort, transition probabilities were not explicitly stratified by age group within the model structure. While age-specific background mortality was incorporated through national life tables to estimate remaining life expectancy, the use of an average-age cohort may not fully capture the heterogeneity of COVID-19 risk across age groups. Given that transition probabilities were derived from observed cohort-level outcomes, the results reflect the actual age distribution of the insured population during the study period rather than a hypothetical uniform-risk cohort. Third, our health-related quality of life and partial recovery state estimates rely on values drawn from international sources due to limited local data. Although the assumptions were conservative and evidence-based, differences in population health preferences may influence QALY estimates. Fourth, the model does not incorporate indirect effects of vaccination, such as reductions in transmission, herd immunity effects, or broader societal and productivity benefits. As a result, the estimated value of vaccination may be conservative from a societal perspective. Fifth, potential adverse events associated with COVID-19 vaccination were not explicitly modelled. However, given the low incidence of severe vaccine-related adverse events relative to COVID-19 morbidity and mortality, their inclusion is unlikely to materially alter the overall conclusions. Sixth, wave and variant confounding is present because vaccination became available only in February 2021. Non-vaccinated individuals accumulated exposure during the wild type and alpha dominated earlier waves, while vaccinated individuals faced delta and omicron variants. Data suggest that alpha and delta were more severe than wild type, while omicron was the least severe variant [49]. This bias may favour vaccination on balance, and our results may overstate the actual cost savings attributable to vaccination. Nevertheless, the magnitude and consistency of the cost savings observed across all outcomes, strategies, and discount rates suggest that vaccination would remain cost-saving even after accounting for these biases, though the absolute magnitude of savings might be lower.

Overall, this study adds context-specific, real-world evidence on the economic value of COVID-19 vaccination within a South African private health insurance setting. The strong performance of both partial and full vaccination strategies, with partial vaccination being dominant and full vaccination being cost-saving at base case and in the majority of simulations, points to the impact vaccination had in reducing disease severity and healthcare expenditure. These findings contribute to the growing empirical literature on the economic value of vaccination programmes in middle-income settings.

## Conclusion

This economic evaluation, using real-world health insurance claims data from 1 March 2020 to 30 December 2022, provides evidence that COVID-19 vaccination was associated with improved health outcomes within a South African privately insured population. From a third-party payer perspective, both partial and full vaccination strategies resulted in lower lifetime healthcare costs and gains in life years and quality-adjusted life years compared to non-vaccination, with partial vaccination also being cost-saving. However, the second dose (full vs. partial vaccination) was not cost-effective relative to the South African threshold in this young, lower‑risk population, with an ICUR of $64,424 per QALY gained.

These findings indicate that funding initial vaccination during the pandemic represented an efficient allocation of healthcare resources within the insured sector, but that the marginal value of the second dose was limited in this demographic. Caution should be exercised in generalising these findings to populations with different demographic and risk profiles.

## Data Availability

The datasets used and/or analysed during the current study are available from the corresponding author on reasonable request.
